# Acceptability of serosurveys in southern Zambia: data collector and caregiver perspectives

**DOI:** 10.1007/s44155-023-00032-6

**Published:** 2023-02-20

**Authors:** Andrea C. Carcelen, Rupali J. Limaye, Simon Mutembo, Mutinta Hamahuwa, Philip E. Thuma, William J. Moss, Kyla Hayford

**Affiliations:** 1 Department of International Health, Johns Hopkins Bloomberg School of Public Health, 415 N. Washington Street, Floor 5, Baltimore, MD 21231 USA; 2 Department of Epidemiology, Johns Hopkins Bloomberg School of Public Health, Baltimore, MD USA; 3 Government of the Republic of Zambia, Ministry of Health, Lusaka, Zambia; 4 Macha Research Trust, Choma, Zambia

**Keywords:** Acceptability, Serosurvey, Specimen collection

## Abstract

**Background:**

Factors associated with whether individuals choose to participate in serosurveys are not well understood. Understanding perceptions from multiple perspectives, including the perspectives of both data collectors and participants, through a holistic model such as the socio-ecological model contextualizes individual, interpersonal, and structural level influences on survey research participation. We used a multiple methods approach to characterize reasons for serosurvey participation in communities in Southern Province, Zambia where a serosurvey was conducted in 2016.

**Methods:**

The first phase conducted focus group discussions and in-depth interviews with 24 data collectors who participated in a measles-rubella serosurvey in 2016. The second phase surveyed 34 caregivers at health facilities to identify barriers and facilitators to serosurvey participation. Emergent themes were then classified into a socio-ecological model using individual, interpersonal, and structural level constructs.

**Results:**

Common themes emerged from data collectors as well as caregivers surveyed. At the individual level, providing incentives was a facilitator, and some religious beliefs were described as a barrier to serosurvey participation. At the interpersonal level, family dynamics and community peer influences could help or hinder serosurvey participation. Community health workers were consistently named as facilitators of participation. At the structural level, concerns about specimen collection, who was selected for serosurveys, and not receiving test results arose as potential barriers. The most frequently reported facilitator was provision of information about the purpose of the serosurvey (85% of respondents). The most frequently reported barrier was lack of clarity regarding use of their blood specimen (53% of respondents). For specimen collection type, caregivers consistently preferred finger prick blood collection over both venous blood draw and oral swabs.

**Conclusion:**

Serosurvey participation was deemed acceptable to most study participants. The socio-ecological model revealed barriers and facilitators for participation to guide strategies to improve participation which can be applied to ongoing serosurveys for SARS-CoV-2. Serosurveys should continue to develop engagement plans to provide information about blood collection ahead of the serosurvey and communicate the objectives of such studies through trusted sources such as community health workers and traditional leaders.

## Background

The validity of serosurveys in health research relies on recruitment of a representative population. However, factors associated with whether individuals choose to participate in serosurveys are not well understood. Reasons for participation in health research more broadly include direct individual benefits [1], altruistic reasons, community benefits, and a feeling of accomplishment by contributing to research [2].

Conceptual models, such as the health belief model, active community engagement continuum and the socio-ecological model, have been used to identify influences of health-related behaviors [3, 4]. The socio-ecological model posits that people’s behavior is determined not just by individual-level aspects but also by intrapersonal factors, interpersonal processes, institutional factors, community factors, and public policy [5]. This model can be applied to understand participation in health research, including serosurveys and the influence of social norms, community as well as religious leaders [6].

Serosurveys require additional resources for social mobilization because biological specimens, often blood, are collected from participants [7]. Refusal rates tend to be higher for surveys that collect blood specimens than surveys that collect only questionnaire data due to potential discomfort, added time for specimen collection, or participants not trusting what is done with their blood [8]. To monitor how non-participation could introduce bias, refusal rates for serosurvey participation are tracked and reported, as those who participate may be different than those who refuse. However, there is limited data explaining why individuals refuse to participate in serosurveys in sub-Saharan Africa [6]. To improve participation in serosurveys, better understanding is needed on how communities perceive serosurveys. Understanding perceptions from multiple perspectives, including the perspectives of both data collectors and participants, can provide a more comprehensive view on what influences participation and subsequently inform ways to improve participation [9].

To explore this, we conducted a study conducted in Southern Province, where at least two serosurveys were conducted in 2016. Southern province is the fourth most populous province with an estimated HIV prevalence of 13% among adults, and first dose measles vaccination coverage of 86% [10, 11]. In early 2016, an HIV incidence and prevalence survey, ZamPHIA, was conducted by collecting venous blood as a household-based survey; with an 11% refusal rate [12]. In late 2016, a serosurvey to assess measles and rubella seroprevalence as part of a vaccination coverage survey was conducted. This serosurvey collected dried blood spots from participants and included a sensitization plan to inform key stakeholders of the serosurvey [13]. Data collectors worked with community health workers from the local clinics, but there was still a 12% refusal rate [14]. We used a multiple methods approach to better understand and characterize reasons for serosurvey participation from both the data collector and caregiver perspectives.

## Methods

### Study design

We used a convergent multiple methods design in which qualitative and quantitative data were collected sequentially, analyzed simultaneously, and interpreted together. The study was conducted in two phases in Southern Province. The first phase was conducted immediately following the measles-rubella serosurvey in December 2016. We conducted in-depth interviews and focus group discussions with data collectors to evaluate implementation of the serosurvey and their perception of community acceptability. The second phase was a cross-sectional survey conducted in April 2017 among caregivers at health facilities. It consisted of questions related to what would motivate or hinder participation in a serosurvey.

### Participants and data collection

In the first phase, data collectors who were part of the measles and rubella serosurvey teams were purposively sampled to include data collectors who had different roles on the team. Data collectors were recruited during the serosurvey and invited to participate immediately after the serosurvey. Eleven supervisors were interviewed individually, and 2 focus group discussions (FGD) for data collectors were conducted for the 13 data collectors, facilitated by a local qualitative researcher from Macha Research Trust who was external to the serosurvey. Verbal consent was obtained from all participants. Interviews and FGD guides included questions on how data collectors were received by the community and perceptions of community acceptability of blood collection. Interviews and FGDs were conducted in English, recorded, and transcribed.

For the second phase, a convenience sample of 34 caregivers who happened to be attending health facilities during our 2-week period of data collection at nine different health centers across three districts in Southern Province, Zambia was selected. These districts had also participated in the post-campaign coverage survey and serosurvey in 2016. Caregivers present at the health facility were approached for recruitment by a healthcare worker in the waiting room. Participants were not required to have participated in a serosurvey, but the facilities were in areas which were eligible to participate in the previous serosurveys. Caregivers provided verbal consent and were asked questions related to the acceptability of blood collection in the community, serosurvey participation, barriers and facilitators to serosurvey participation, specimen preference, and self-efficacy. Questionnaires were administered in Tonga by a trained local collaborator from the Ministry of Health in a private area in the health facility.

Ethical and regulatory approval was obtained from the institutional review boards at Johns Hopkins University School of Public Health and Macha Research Trust, and the Zambia National Health Research Authority.

### Data analysis

Qualitative data from the first phase were analyzed using a grounded theory approach in Atlas TI (version 10.1). Grounded theory allows the data to speak for itself without using a priori assumptions [15]. There were several rounds of codebook development and coding, including initial open coding to generate categories, followed by axial coding to relate these categories, and finally grouping by theory [16]. Coding mismatches between two analysts were discussed until a resolution was reached. The constant comparison approach was used to generate themes through simultaneous coding and analysis [15]. Preliminary analysis of themes from the first phase informed the survey questionnaire for caregivers in the second phase.

Quantitative data from the second phase were cleaned and analyzed using STATA 14. Descriptive statistics were performed to examine frequencies, and grounded theory was used to code open-ended questions in Excel. Quantitative and qualitative data were analyzed together, classifying emergent themes into a modified socio-ecological model as a framework using individual, interpersonal, and structural level constructs.

Results from both phases were categorized by acceptability factors that affect participation in serosurveys, including barriers and facilitators at each socio-ecological model level. 

## Results

### Participant characterization

In the first phase of data collection, there was male representation as well as multiple districts, teams and roles on the teams included (Table 1).

**Table 1:** Characteristics of data collectors who participated in in-depth interviews and focus group discussions

Data collector characteristics	n = 24	%
Role		
Supervisors	11	45.8
Data collectors	13	54.2
Male	8	33.3
Districts covered per team		
Kazungula, Livingstone	6	25.0
Choma, Kalomo, Namwala	5	20.8
Choma, Pemba	4	16.7
Gwembe, Monze	6	25.0
Province level	3	12.5

In the second phase, all 34 caregivers surveyed were female. Most of the caregivers were younger than 30 years of age (68%). The most prominent religion was Seventh Day Adventists (38%) (Table 2). Almost half of those surveyed reported having traveled for longer than one hour to arrive at the health facility. Most of the caregivers were at the facility for their children to be vaccinated, and 91% had children who were under 5-years of age.

**Table 2:** Socio-demographic factors and serosurvey response characteristics of caregivers who participated in the survey

Caregiver characteristics	n = 34	%
District		
Namwala	12	35.3
Kazangula	11	32.4
Choma	11	32.4
Religion		
Catholic	7	20.6
Pentecostal	3	8.82
Protestant	4	11.8
Seventh day adventist	13	38.2
Other	7	20.6
Time to vaccination clinic		
< 30 min	8	23.5
30–59 min	10	29.4
60 + minutes	16	47.1
Who influences decision making*		
Self (caregiver themselves)	23	67.65
Family	14	41.18
Healthcare worker	5	14.71
Community health worker	2	5.88
Chief or leader	0	0
Received information from CHWs		
Yes	13	38.2
Type of information:		
Vaccination	8	61.5
Measles	2	15.4
Nutrition	2	15.4
Other	1	7.7
Participated in surveys		
Yes	10	29.4
Type of survey^		
HIV	4	40.0
Malaria	5	50.0
Sanitation	1	10.0
Doesn't remember	2	20.0
Bodily fluid collected^	8	80.0
Results received	5	62.5
Number of surveys in which participated^		
0	1	10.0
1	6	60.0
2–4	3	30.0
Willingness to participate in future serosurveys		
Willing to allow finger prick	25	73.5
Willing to allow child’s finger prick	21	61.8

(*)Participants could name multiple sources of influence. (^)Type of survey, whether bodily fluid collected and number of surveys in which participated was only asked of the participants who answered “yes” to previous survey participation

Ten caregivers (29%) reported having participated in a household-based survey in the past. These included surveys for different health purposes including malaria, HIV, and sanitation. Of those, eight caregivers (80%) reported that the surveys collected specimens, about half (50%) received their test results, and three caregivers (30%) participated in more than one household survey. When asked directly if they would allow finger prick blood collection at their households, 74% accepted for themselves, and 62% said they would allow their children to participate.

**Fig. 1:**
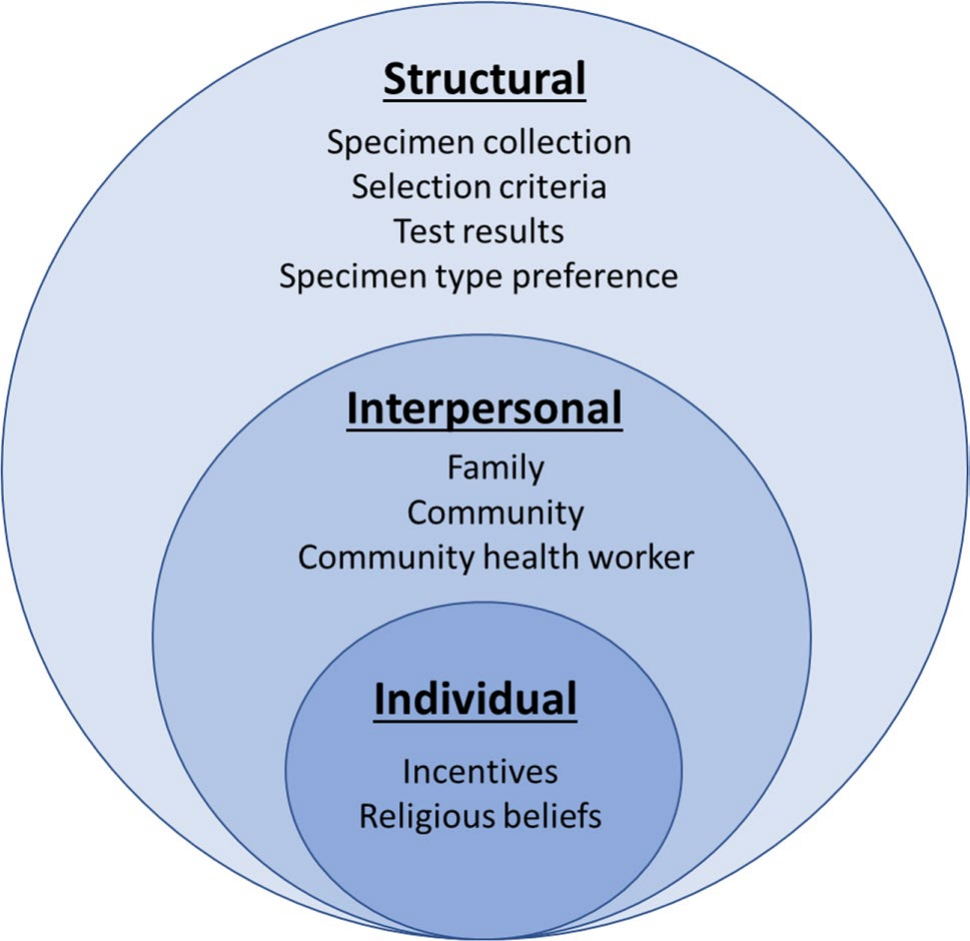
Emerging themes related to acceptability of a serosurvey within a socio-ecological model

The acceptability themes that came out of our findings are summarized by socio-ecological model level in Fig. 1 and presented below. Individual influences included personal motivation such as incentives and religious beliefs. Interpersonal influences affecting participation in serosurvey decision making included community, family structure, and the community health worker influence on participants. Structural influences included factors associated with survey implementation. These included questions related to specimen collection, including specimen preference type, who is asked to participate in serosurveys (i.e. sampling), and provision of test results.

### Acceptability: individual influences

Caregivers listed individual level facilitators that encouraged serosurvey participation, including personal desire to know more about health issues (29%) and altruistic notions (3%) (Table 3). Data collectors noted participants wanted compensation, either monetary or consumption goods, for their participation. Religious beliefs were mentioned as a barrier to specimen collection by both the caregivers and the data collectors.

**Table 3:** Individual influences: barriers & facilitators to serosurvey participation according to caregivers (n = 34)*

Barriers and facilitators to serosurvey participation	n	%
Barriers		
Unsure what is being done with blood	18	52.9
Religious beliefs	15	44.1
Scared to know results	14	41.2
Not getting test results	4	11.8
Fear of needles	4	11.8
Perceived stigma for getting tested	3	8.8
Perceived satanic practices in the end use of collected blood	2	5.9
Safety concerns with blood collection	2	5.9
Research fatigue	1	2.9
Facilitators		
Information about study provided	29	85.3
Have people come to central location for serosurvey	16	47.1
Someone community trusts (like CHW)	10	29.4
Desire to know about health issues	10	29.4
Community thinks they will get a benefit	4	11.8
Go house to house for serosurvey	1	2.9
Desire to better society	1	2.9

(*)Participants could name multiple barriers and facilitators. Totals do not sum to 100%

#### Incentives

Data collectors identified compensation as a specific individual level incentive. One data collector recalled that monetary payment was not provided to avoid coercion, as this would be unethical:

*We discussed (incentives) in workshop. That it would look as if we were paying for blood, but people really wanted to be compensated for their blood.* - FGD 1, serosurvey data collector 5

Data collectors noted that, participants requested other forms of compensation:

*On the same collecting blood, others were saying, ‘you should compensate us’. Simple stuff like a drink, milk. That’s what normally happens when others go out to collect blood. So why are you not compensating us?* - FGD 1, serosurvey data collector 2

#### Religion

Religious beliefs were primarily described as a barrier to serosurvey participation due to blood collection, as mentioned by 15 caregivers (44%). Zionism, a religious grouping that preferred traditional medicine to biomedicine [17] did not allow provision of blood specimens. Among the caregivers who were Seventh Day Adventists, one-third reported they would not allow blood collection. Two caregivers also mentioned Satanism, the worship of Satan or obsession with evil, as a fear or barrier to blood collection.

Many of the concerns that data collectors reported related to traditional beliefs that blood was used for witchcraft and satanic rituals, as articulated by this data collector:

*You know, the people, they think that when you get blood from them, maybe you want to do rituals on them. That’s what affected mostly the blood collection. And there are some areas where they believe really too much into Satanism.* -IDI, PCES supervisor 2

Data collectors reported being told that they were not welcome to collect blood because community members held the belief that data collectors themselves were Satanists.

When you start explaining to them [why we are here], they get surprised and some were even chasing us, telling us, “Go!”, “Why do you want our blood? You are just Satanists!” -FGD 2, data collector 7

While there were multiple reports of the community calling data collectors Satanists, one supervisor questioned whether Satanism was prevalent in the areas where the serosurvey occurred.

The belief around here was, they were like, maybe you want to…. offer my blood as a sacrifice. Maybe you are Satanists. Yes. They believe in Satanists collecting blood from people.

Facilitator: Does that happen a lot here?

They’ve never even seen it. Neither have I. So [Satanism] is just rumors. -IDI, serosurvey supervisor 2

### Acceptability: interpersonal influences

The decision of whether or not to participate in a serosurvey falls under a broad healthcare decision making umbrella. Survey participants identified several interpersonal influences on healthcare decision making, including themselves, family members, and community health workers (Table 2). Data collectors similarly mentioned family members and community health workers as interpersonal influencers, but also added that community norms and specific community members influenced individual choice to participate. Caregivers also noted the importance of providing information about the objective of the serosurvey (85%) and having information communicated by someone the community trusts, such as the community health worker (29%).

#### Family

Family dynamics played a role in potential serosurvey participation, as noted by 14 caregivers (41%) who cited fathers or grandparents as influencing their healthcare decision making process (Table 2). Data collectors also noted that permission from caregivers to allow children to participate in the serosurvey was challenging in some areas because mothers required permission from the fathers, or even tried to have their children participate but the fathers refused by hiding their children, as told by this data collector:

*The husband said, “no, I cannot do this.” The wife… wanted to participate, but the husband got one of the children [and took] him in the house. Again, [he] called another [child], and sent to get another [child]. The mother was like, “what do I do now?”* -FGD 2, data collector 7

In addition to the need for approval from the child’s father, data collectors stated that women also needed to be present for their child to participate in the serosurvey. This is because mothers have the information to answer the questions regarding vaccination history, as noted by this data collector:

*I guess that made me understand that the men that head the households don’t really have enough information on their children and their children’s health.* -IDI, PCES supervisor 3

Additionally, older children and even young adults followed the decision of their parents as to whether they participated in the serosurvey.

#### Community

For areas where there were refusals, data collectors believed there had been insufficient social mobilization efforts. One data collector thought participants in a community had gathered to make a collective decision on participation in the survey:

*For example, you visit me at this house. You also visit the other house. If I’m not clear, I’ll go and ask my neighbor, ‘did you hear what these people say?’ And sometimes the community will want to meet. ‘Let’s do this when they come, let us all refuse.’ By the time you go, you try here, you try there. Like one village where we went, everyone refused. .* -IDI, serosurvey supervisor 1

Some data collectors reported that communities said they could not participate because the village headmen and local health facilities had not been informed of the survey, suggesting that community leadership served as gatekeepers to serosurvey acceptance:

*Their concerns were…if it’s from the Ministry of Health, they always pass through the clinics, or the health centers/facilities to say or to announce to the community to say if you come on such and such dates to the clinic, there will be this. Yes, they know. So, the villages, the headmen, most of them, they were not aware. So, they were like, we cannot participate because the headman does not know. So, they were all referring to the headman.* -IDI, serosurvey supervisor 2

#### Community health worker

Another influence in the acceptability of serosurvey participation was the community health worker (CHW). Among caregivers, one-third had received health information from community health workers, including information about vaccination (Table 2). CHWs were viewed as important facilitators by both data collectors and caregivers for two primary reasons: they were recognizable by the community, and they were a source of trusted information.

Caregivers articulated that it was important to have someone they recognized on the survey team, as this was an important factor in deciding whether or not to participate in a serosurvey:

*The people doing the surveys must go through the health center and come with people from the health center. Then we know they are real. I would not accept unless you come with someone I know from the health center.* -caregiver 26

Overwhelmingly, data collectors agreed that having a CHW accompany the serosurvey teams was helpful for both knowing where to go in the community as well as sensitization of the community. One data collector noted:

*At the clinic [we picked up] the health worker, and... the community health worker. We used to move with the two of them. [When] we would go to certain households, [the community health worker] was the one who first, started introducing us, so it was a bit easy.* -FGD 2, data collector 4

Almost all caregivers (85%) reported that the provision of information about the purpose of the serosurvey was the primary facilitator to encourage participation (Table 3). This sentiment was echoed by data collectors, who recommended sensitization in the communities ahead of time, as mentioned by this data collector:

*Because most of the people in rural areas are illiterate, so it’s very difficult for them to understand when you go there to explain something. Even if you explain something in depth, they will keep on asking you the same question (for clarity). So if they (CHW) visit them, they explain…maybe it can be easier when other people come in now to do surveys in their areas.* -IDI, PCES supervisor 2

Data collectors believed that providing information in advance about the serosurvey to the communities facilitated implementation in some areas, as noted by another data collector:

*[For] sensitization I think it’s also important to give the correct information to people who are requested to go in the community to sensitize. It would make the work very easy.* -IDI, serosurvey supervisor 1

Community health workers should be the ones to provide the information about the purpose of the serosurveys to improve participation, as highlighted by this data collector:

*I think the best thing to do is, those places...if they can make use of the CHWs to sensitize them. So that when there’s such a program, the people are aware, and they know the reason why people travel from distant places to go to their places to do such programs. Because the level of understanding is what is lacking.* -IDI, PCES supervisor 2

### Acceptability: structural influences

Structural level barriers and facilitators were grouped into issues related to specimen collection, selection criteria for participation, and the results of testing. Structural level influences on participation were related to specimen collection, namely, the amount of blood and where blood was being taken, and the desire to know test results. In the caregiver survey, being unsure of what was being done with the blood (53%) and fear of receiving test results (41%) arose as significant barriers to participation (Table 3). However, data collectors and caregivers both also indicated that receiving test results was an important facilitator to serosurvey participation.

#### Specimen collection

Barriers named by both caregivers and data collectors were primarily centered on issues related to specimen collection. Most caregivers (53%) listed not knowing what was being done with the blood as the primary barrier to participation. Similarly, data collectors mentioned that potential participants asked many questions despite the explanations provided during the consent process: “*Why are you taking our blood? Why are you studying this?”* There were also some questions related to rubella virus, the vaccines, and how antibodies worked.

In the qualitative data, data collectors noted comments about specimen collection related to past experiences with serosurveys. In areas where the community participated in an HIV serosurvey earlier that year, some of the questions were related to their experience with that survey. The primary concern that data collectors noted from the community was “*Where are you taking our blood?”* In terms of where specimen collection should occur, the survey found that half of caregivers thought having people come to a central location, such as a clinic within the community, would facilitate serosurvey participation (Table 3).

#### Selection criteria

Both caregivers and data collectors mentioned concerns about the same communities being repeatedly asked to participate in surveys. One caregiver listed too many researchers coming to collect blood from the community as a barrier (Table 3). This sentiment was echoed by the data collectors, as noted by one:

*Mostly people go there to collect blood. Different programs, from MOH [Ministry of Health]. So every time it’s just blood, blood, blood...* -FGD 1, data collector 5

In the qualitative data, data collectors noted additional concerns from the community as to why certain people were being included in the survey and others excluded. In some cases, this was from participants who were selected for the serosurvey, asking why everyone was not being included. In other cases, it was community members wanting to participate, and asking why they were being excluded. Both experiences were mentioned by this supervisor:

*You would find that the household that you…pick. Those that were picked will say, “why us? Why are you not getting everyone?” So sometimes you would find that those that are left out, like, “why have you left us?” There was someone [who] got upset. “We’ve left our work. We’ve stayed [home]. We stopped what we were doing, and…you are not going to include us.”* -IDI, serosurvey supervisor 1

Similarly, data collectors said community members from rural areas asked why they were targeted, when people in urban areas were not.

*…Others were saying why is it that you always come in the rural areas? When we phone our relatives in town, they say they don’t experience such things.* -FGD 1, data collector 3

There was also a question about why older individuals were being included, when they had not received the vaccine, as mentioned by this data collector:

*Then the other question was, why are you selecting households, not collecting blood from everyone who was given an injection. Or that vaccine you are saying. And why are you collecting blood from everyone, including the oldest, when they didn’t receive those vaccines.* -IDI, serosurvey supervisor 2

These concerns about equity of selection did not arise in the caregiver survey.

#### Test results

Providing test results was viewed as both a barrier and a facilitator. In the caregiver survey, fear of test results because they fear being told they have a disease was a substantial barrier to serosurvey participation (41%), yet all said receiving their test results was important to them. A few caregivers said that not receiving their test results was a barrier to serosurvey participation (12%). Additionally, 79% stated they were willing to accept summary, community-level results provided to the district health officer rather than individual test results.

Just as test results were reported as important to caregivers, data collectors also reported the most commonly referenced requests from the community was wanting to know the results of the testing being done on their blood. Data collectors reported complaints from the community that data collectors in the past collected blood from them but did not provide test results. As noted by one data collector from the serosurvey:

*After sensitizing them, they gathered to refuse…say[ing] ‘there are a lot of people coming to take our blood. They don’t come back to tell us our results in person.’ Everyone wants to know their results. Sometimes you say after pricking [you will get results], they say ‘no, give us our blood back.’ They say ‘you are doing for everyone in this community. We want our results. You just come, you don’t bring us anything.’* -FGD 1, data collector 6

A data collector also reported the community was eager to receive their test results:

*I think the community is also anxious to know their results. Because most of the time, these studies are being done, but the response is not taken back to the community.* -IDI, serosurvey coordinator

One data collector also specifically noted that not receiving test results negatively affected the community’s willingness to participate in the study:

*The biggest question that we faced was…most of the time, we go to them to collect blood, but we don’t tell them the outcome. When we collect blood from them, when we go, we [leave forever], without even informing them what we have found. So now they were like, ‘you people, us we are very much willing to participate in this program. But the problem that we have is every time you come, you collect our blood, you go. But when it comes to giving us the results, you don’t even tell us what you find.’ That was their main concern.* -IDI, PCES supervisor 2

Although HIV testing was not specifically asked in either phase, there were reports of participants wanting to be tested for HIV as well as refusals to participate in serosurveys for fear that they would be tested for HIV. Almost half of participants feared learning their test results, and some thought there could be stigma related to having their blood collected (9%) (Table 3). While not explicitly stated by all, there were undertones of HIV testing in the open-ended responses. One caregiver mentioned long-term treatment despite lack of symptoms, and another caregiver specifically stated fear of stigma about HIV if they participated. Similarly, data collectors also mentioned that people thought blood was being collected for HIV testing.

Finally, the data collectors also mentioned privacy as a concern from participants who feared their test results would become known. Participants pointed out that if the survey was anonymous, then names should not be required for participation.

#### Specimen preference

**Fig. 2:**
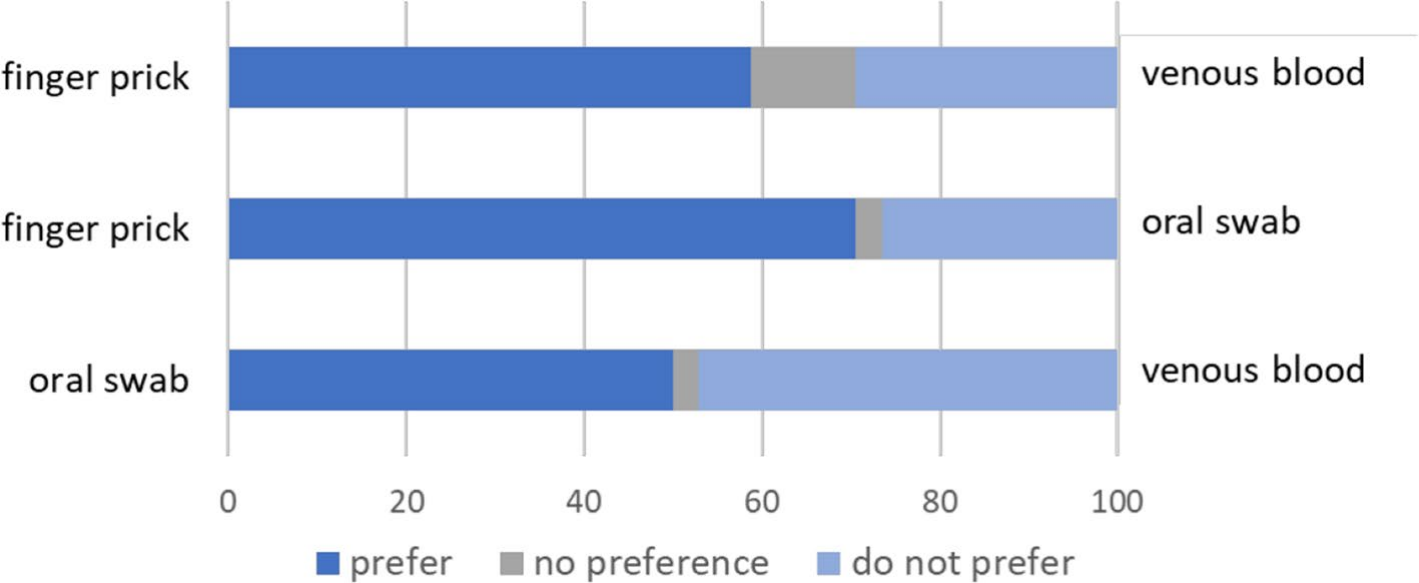
Pairwise specimen preference among caregivers comparing oral swab, venous blood and finger prick collection methods. (n = 34) Participants were asked pair-wise specimen preference types, and stated either: preferred, no preference, or did not prefer one specimen over another. Only one response allowed per pairwise comparison

Specimen preference was specifically asked in the caregiver survey only. The survey demonstrated that participants tended to prefer finger prick blood collection over venous blood draw (59%) and finger prick blood collection over oral swabs (71%); however, there did not seem to be a strong preference between venous blood draw and oral swabs (Fig. 2).

General reasons for specimen preference included simplicity of the procedure (24%), procedure that caused least pain (24%), a desire to know their test results (21%), and choosing a specimen based on the doctor’s recommendation (15%) (Table 4). One-quarter of participants believed it was easier to have blood taken than saliva, while a few (9%) thought saliva was easier to collect. This could in part be due to participants saying they had never heard of testing from saliva. As noted by a caregiver, they knew diseases could be detected in blood:

*The doctor can detect a lot of diseases in blood, but I don't know if he can detect anything in saliva.* -caregiver 27

**Table 4:** Reasons caregivers preferred a specimen type (n = 34)

Reason for preference*	n	%
Simplicity of procedure	8	23.5
Pain from procedure	8	23.5
Desire to know test results	7	20.6
Doctor recommendation	5	14.7
Amount of blood	4	11.8
Blood is better than saliva	9	26.5
Saliva is better than blood	3	8.8
Never heard of saliva testing	5	14.7

(*) Participants could provide multiple reasons for their specimen preference

Similarly, another participant noted that they had experience with blood draws and finger pricks:

*I have never had a mouth swab before so I wouldn’t know how it works. I am familiar with a blood draw or pricks*. -caregiver 24

Overall, caregivers mentioned more familiarity with blood draws and finger pricks than with oral swabs.

## Discussion

This study characterizes barriers and facilitators to participation in serosurveys. We provide two perspectives on the acceptability of serosurveys: that of data collectors and caregivers as potential serosurvey participants. As demonstrated by the socio-ecological model, individual level factors were not the direct driving facilitators to participation, but rather interpersonal factors, and most importantly, structural issues related to serosurvey implementation drove participation indirectly.

At the interpersonal level, community healthcare workers were an important driver to participation because they provided information about the serosurvey and were trusted by the community. Family dynamics also came into play, as caregivers noted they made decisions with other family members, and data collectors noted that sometimes the father had to agree for the child to participate in the serosurvey. Including their perspective in future research would be important. At the structural level, understanding use of specimen collection and provision of test results affected participation. Capitalizing on the facilitators can help frame messaging for communities to improve participation, which can be applied to future serosurveys such as those for SARS-CoV-2.

We noted differences in themes at the interpersonal and structural levels. Caregivers said that they were the most significant decision makers; whereas data collectors thought religious and community leaders were a major influencer in the acceptability of the serosurvey. At the structural level, questions about equity of participation arose from data collectors in terms of who was being chosen to participate in the serosurvey, with some groups feeling targeted and others excluded. There were also privacy concerns about test results becoming known. These likely did not arise in the caregiver survey because people were not actually being asked to participate in a serosurvey. Privacy concerns have been noted for other studies that require dried blood spot collection [18]. Religion was noted as an important individual-level barrier, community health workers as a major interpersonal-level facilitator, and questions about specimen collection and not receiving test results as potential barriers at the structural level. Whether HIV testing was being performed was also mentioned as both a facilitator and a barrier from both perspectives.

It was uniformly agreed that it was important for participating communities to receive test results, either individual results or community-level results. Providing community-level results, such as during community meetings, would help overcome the logistical challenges of providing individual test results. While there were some contradictions in that participants feared receiving test results if they did not feel sick, this seemed to be more with regards to HIV rather than measles and rubella testing. Provision of test results has been noted as a factor for participation in blood draws in clinical trials [1]. In HIV/AIDS, malaria, and SARS-CoV-2 research, point-of-care tests are available, so results can be provided immediately. These tests indicate current infection. However, for measles and rubella this is not yet an option.

Although our focus was on the acceptability of participation in serosurveys, some issues arose that may be applicable to all household surveys. Issues specific to serosurveys were religious beliefs barring specimen collection at the individual level, and provision of test results and questions about specimen collection at the structural level. Lack of knowledge about what would be done with blood specimens is a common barrier to research participation [19]. As noted by caregivers, providing information to the community would overcome questions about specimen collection. This has been done for some groups through community education, including engagement of religious leaders [20]. Understanding how much dropout occurs when blood collection is added to the survey, as compared to what dropout would have been without blood collection, is an important consideration for serosurveys.

In terms of specimen preference type, finger prick blood collection was preferred by caregivers. This finding is consistent with another study in Zambia that also reported preference for finger prick over venous blood collection by both participants and providers [21]. By contrast, a study in Tanzania found that community members preferred venipuncture over finger prick blood collection [22]. Our study results indicated that blood was preferred. Reasons for this included participants believing it is an effective means of detecting disease, whereas collection and testing saliva was unfamiliar to most participants. This study population opinions are likely influenced by the fact that there are programs and research studies monitoring HIV/AIDS and malaria in the area [12, 23]. Participants may be accustomed to finger pricks for rapid testing. Although this study was conducted prior to the COVID-19 pandemic, serosurveys considering oral or nasopharyngeal swabs for SARS-CoV-2 may need to consider the community’s preference for finger prick specimen collection.

Although we provide the community perspective by surveying caregivers directly, we asked theoretical questions about participation and did not require that respondents had participated in a serosurvey. While 30% of respondents reported participating in a serosurvey, we did not note a difference in the responses between those who had versus those who had not previously participated in serosurveys. Because caregivers were recruited at health facilities, the sample may be biased toward people who are more willing to accept specimen collection because they are health-seeking individuals. Furthermore, most data collectors and all caregivers were female, limiting the perspective from male caregivers despite their influence on the decision to participate in serosurvey. There was a prominent undertone of HIV/AIDS concerns underlying the responses in this population, such as fear of their ‘status’. Whether this is due to direct association with blood specimen collection, previous serosurveys for HIV, or social mobilization campaigns is not known. These results reflect a setting with HIV and malaria transmission, which is accustomed to blood collection for diagnostic purposes, so our findings may not be generalizable to settings where fingerpick blood collection is less common. Because these data were collected prior to the COVID-19 pandemic, it is unknown if acceptance for oral swabs may have changed.

## Conclusions

This paper successfully characterised the barriers and facilitators for community participation in serosurveys in Southern Zambia. Overall, serosurvey participation was deemed acceptable to most study participants. Participants have a right to refuse but these refusals can be minimized by understanding the barriers and facilitators to prevent high refusal rates. The socio-ecological model reveals barriers and facilitators for participation to guide strategies to improve participation. Lessons that could be applied to other serosurveys like SARS-COV-2 and generalised to similar rural settings include continued engagement plans to provide information about blood collection ahead of the serosurvey and communicate the objectives of the study through a trusted source such as community health workers. Considering the number of SARS-CoV-2 serosurveys being done, further research could be done to see if the perspective on specimen collection method has changed.

## Data Availability

The datasets used and/or analyzed during the current study are available from the corresponding author on reasonable request.

## Abbreviations

CHW: Community health worker
FGD: Focus group discussion
HIV: Human Immunodeficiency Virus
